# Beyond small-scale spatial skills: Navigation skills and geoscience education

**DOI:** 10.1186/s41235-019-0167-2

**Published:** 2019-06-13

**Authors:** Alina Nazareth, Nora S. Newcombe, Thomas F. Shipley, Mia Velazquez, Steven M. Weisberg

**Affiliations:** 1 Temple University, 1701 N. 13th Street, Philadelphia, PA 19122 USA; 2 Center for Cognitive Neuroscience, University of Pennsylvania, Philadelphia, PA 19104 USA

**Keywords:** Spatial cognition, Virtual environment, Navigation, Geology, Geographic Information Systems

## Abstract

**Background:**

Research examining the relation between spatial skills and the science, technology, engineering and mathematics (STEM) fields has focused on small-scale spatial skills, even though some STEM disciplines—particularly the geography and geoscience (GEO) fields—involve large-scale spatial thinking at the core of their professional training. In Study 1, we compared large-scale navigation skills of experienced geologists with those of experienced psychologists, using a novel virtual navigation paradigm as an objective measure of navigation skills. In Study 2, we conducted a longitudinal study with novice Geographic Information Systems (GIS) students to investigate baseline navigational competence and improvement over the course of an academic semester.

**Results:**

In Study 1, we found that geologists demonstrated higher navigational competence and were more likely to be categorized as integrating separate routes, compared to their non-STEM counterparts. In Study 2, novice GIS students showed superior baseline navigational competence compared to non-STEM students, as well as better spatial working memory and small-scale mental rotation skills, indicating self-selection. In addition, GIS students’ spatial skills improved more over the course of a semester than those of non-STEM students.

**Conclusions:**

Our findings highlight the importance of large-scale spatial thinking for enrollment and success in the GEO fields but likely also across the broader range of thinking involving spatial distributions. We discuss the potential of GIS tools to develop spatial skills at an early age.

## Significance

People with strong spatial skills are more likely to pursue and succeed in science, technology, engineering and mathematics (STEM) careers. Fortunately, spatial skills are malleable, and improvements in them are both durable and generalizable (Uttal et al., 2013). Improving spatial skills during educational training may be an effective way of increasing a gender-balanced STEM workforce. However, there are two gaps in current knowledge, which the current research addresses. First, existing research focuses almost exclusively on small-scale spatial skills, leaving a gap in our understanding of the relation between large-scale navigation skills and STEM learning, specifically related to the Geography and Geoscience (GEO) fields. Using a large-scale virtual environment, we tested the navigation skills of expert geologists and compared their performance with that of expert psychologists. Second, there is a need for a sustainable spatial training plan that can be integrated into classrooms; one possibility is the integration of Geographic Information Systems (GIS) tools in STEM teaching. The current project tested the effectiveness of GIS training in improving spatial skills in novice students.

## Background

Humans need spatial skills to survive and function in a spatial world: to navigate from point A to point B, to manipulate objects and to invent tools. Strong spatial skills also predict interest and success in science, technology, engineering, and mathematics (STEM) disciplines (Kell, Lubinski, Benbow, & Steiger, 2013; Shea, Lubinski, & Benbow, 2001; Wai, Lubinski, & Benbow, 2009). However, these studies involve paper-and-pencil assessments of “small-scale” object-based manipulations such as mental rotation. Decades of behavioral research, and more recent findings from neuroscience, suggest that spatial thinking is a multidimensional construct involving different cognitive mechanisms and distinct neural networks for dealing with the space of objects (small scale) or environments (large scale; Aguirre & D’Esposito, 1999; Morris & Parslow, 2004; Philbeck, Behrmann, Black, & Ebert, 2000). Thus, an important dimension to consider when investigating the relation between STEM learning and spatial skills is scale. An important gap in our understanding of the reciprocal relation between spatial skills and STEM success involves whether large-scale spatial skills, like small-scale skills, predict enrollment in STEM fields and contribute to STEM success. Do good navigators make good scientists? Do good scientists develop navigationally relevant skills?

Traditional attempts to define and categorize spatial ability into constituent spatial skills (Carroll, 1993; Eliot, 1987; Linn & Petersen, 1985) have not highlighted the scale distinction, likely because psychometric tests did not really tap into large-scale skills. Montello (1993) discusses the importance of scale in understanding psychological spaces and defines four classes of psychological spaces based on the *projective* and not the absolute size of space relative to the human body—figural, vista, environmental and geographical. Small-scale spatial skills are needed at the figural (e.g., a small object) and vista (e.g., a single room) scales, where an individual can visually observe all spatial characteristics with minimal movement (i.e., from a single vantage point). However, large-scale spatial thinking comes into play at the environmental (e.g., a city) scale, where an individual may obtain information about the spatial properties of the space through considerable locomotion and at the geographical scale (e.g., a country), where direct locomotion must be replaced by symbolic learning from maps and models in order to obtain spatial information about the space. If large-scale navigation skills are indeed relevant for success in STEM fields, they should be most relevant in fields that require spatial reasoning on a large scale. The core of professional training in the GEO STEM disciplines (we use GEO to encompass the geography and geoscience disciplines that focus on spatial patterns and include geology, geography, geographic information systems, geophysics, oceanography and atmospheric science, among others) is engagement in spatial encoding and transformation on an environmental and geographical scale. Thus, GEO disciplines may rely on and hone large-scale thinking, which may not be the case in STEM fields like chemistry and physics or even engineering. So far, the disproportionate focus on small-scale spatial skills and their relation to general STEM learning ignores the heterogeneity of both spatial skills as well as that of STEM training.

**Fig. 1:**
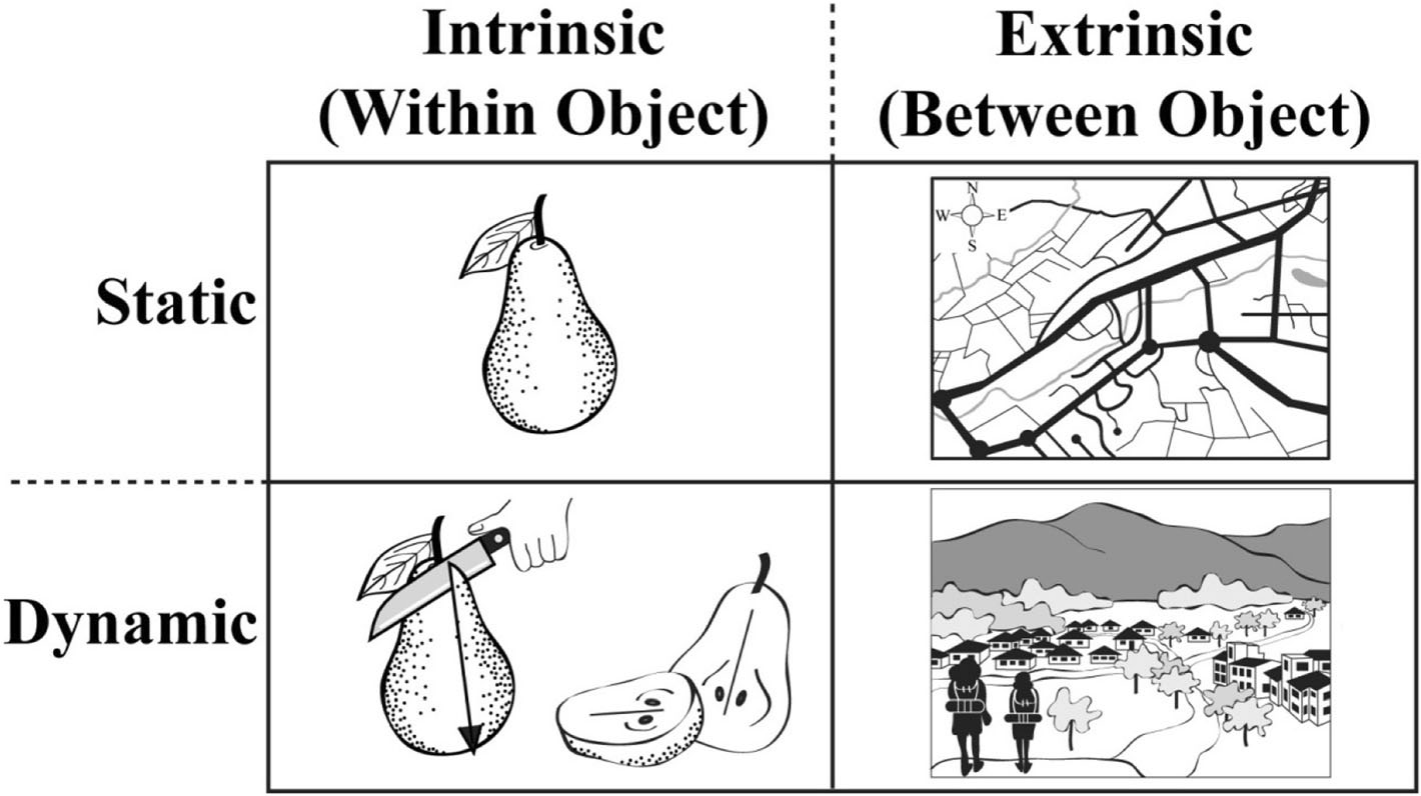
Categorization of spatial skills by intrinsic/extrinsic object information and static/dynamic movement information

A more nuanced approach motivated by findings from neuroscience and psychology (Chatterjee, 2008) is to categorize spatial skills based on the use of intrinsic/extrinsic object information and static/dynamic movement information, as illustrated in Fig. 1 (Newcombe, 2018; Newcombe & Shipley, 2015; Uttal et al., 2013). Common spatial measures used to test spatial skill in the laboratory exist in each of the four quadrants. For example, the small-scale spatial skill of mental rotation involves movement (dynamic) of a single object (intrinsic), whereas the large-scale skill of navigation frequently involves movement (dynamic) of oneself with respect to a set of objects (extrinsic). Research on skills in the extrinsic–dynamic cell at the bottom right has been largely empty.

One of the main reasons for the gap in extrinsic–dynamic spatial research is the challenge associated with conducting lengthy and standardized real-world navigation experiments (Choi, McKillop, Ward & L’Hirondelle, 2006; Heth, Cornell & Alberts, 1997; Holscher, Tenbrink & Wiener, 2011; Ishikawa & Montello, 2006; Schinazi, Nardi, Newcombe, Shipley, & Epstein, 2013). Virtual environment navigation paradigms overcome this challenge. One paradigm—Virtual Silcton—has been used with hundreds of participants of varying ages (Blacker, Weisberg, Newcombe, & Courtney, 2017; Galati, Weisberg, Newcombe, & Avraamides, 2017; Nazareth, Weisberg, Margulis & Newcombe, 2018; Weisberg & Newcombe, 2016; Weisberg, Schinazi, Newcombe, Shipley, & Epstein, 2014). In Virtual Silcton, participants explore a virtual environment modeled along the lines of a college campus comprising two main routes and two connecting routes. Participants use computer arrow keys to navigate through the virtual world and to learn the names and locations of target buildings along the two main routes. Using this methodological tool, participants exhibit large and robust individual differences in their navigation skills. Both adults and children can be categorized into three distinct navigator types: integrators, non-integrators and imprecise navigators. Integrators can connect different routes to create a cognitive map of the environment; non-integrators can represent independent routes but are unable to relate them to each other; and imprecise navigators have trouble even representing independent routes. With this new tool, we can now ask the question of whether large-scale spatial skills contribute to STEM success.

In this article, we aim to address the gap in the spatial and STEM literature by conducting two studies of GEO disciplines to evaluate the relation between navigation skills and experience with these kinds of science. In two studies, we investigate individuals who differ in their mode of learning and levels of domain expertise but have visualization and manipulation of environmental and geographical spaces at the core of their training. There were two approaches, involving two designs with two different disciplines at two different levels of expertise. In Study 1, we investigated large-scale navigation skills in experienced solid earth geologists—individuals who have acquired a master’s or Ph.D. degree in geology accompanied by field experience—in contrast with psychologists with equivalent years of experience in that field. In Study 2, we examined self-selection and learning in undergraduates taking a GIS course in contrast to those selecting a nonspatial course.

## Study 1

Geology is the study of observable large-scale environmental features to deduce natural events that may have occurred over a period of millions of years. Geologists study physical processes that span large-scale spatial relations of tectonic plates to more microscopic phenomenon like the spatial organization of mineral grains (Hegarty, Crookes, Dara-Abrams, & Shipley, 2010; Kastens, Agrawal, & Liben, 2008; Kastens et al., 2009). Thus, one would expect that an experienced geologist is comfortable making judgments pertaining to spatial pattern detection and transformation associated with geological events. However, do these domain-specific skills extend to spatial skills? Previous research has established a connection between small-scale spatial skills and geology training (Kali & Orion, 1996; Orion, Ben-Chaim, & Kali, 1997; Piburn et al., 2005). In a recent study, Hambrick et al. (2012) studied 67 geologists with varying levels of experience who completed a realistic bedrock-mapping task along with a battery of cognitive ability tasks. The authors found that visuospatial ability predicted performance on the bedrock-mapping task for novice but not experienced geologists, suggesting that high domain knowledge may sometimes allow the circumvention of domain-general cognitive limitations in domain-specific tasks.

In an initial study of large-scale spatial skills, Hegarty et al. (2010) used an online questionnaire to collect self-reports on spatial skills at different scales from 796 scientists and specialists in different disciplines. They found that geoscientists (here, the term geoscientists refers to specialists in geology, oceanography and meteorology, but does not include geography or GIS) reported the highest levels of navigational competence and confidence as measured by the Santa Barbara Sense of Direction Scale (SBSOD). Geographers were a close second. Other scientists reported lower skill levels. Although self-reported navigation correlates with objective measures of navigation behavior (e.g., Weisberg & Newcombe, 2016; Weisberg et al., 2014), it is an indirect measure of ability and it is important to determine whether geologists do indeed have better navigation skills.

In Study 1, we anticipated that our sample of experienced geologists would point more accurately between different points in the environment and be more likely to be categorized as integrators, demonstrating higher navigational competence as compared to experienced psychologists. Thus, Study 1 addresses the gap in spatial skills literature by directly linking large-scale navigation skills to the GEO fields and taking the claim beyond self-reporting.

## Method

### Participants

A total of 28 experienced geologists (12 female; mean (*M*) age = 40.7 years, standard deviation (*SD*) = 9.7) were recruited via email as well as in person at geology-centered conferences with the goal of collecting as much data as possible over a period of 1 year. The majority of geologists (*n* = 20) held a Ph.D. at the time of participation in this study. Data were also collected from geologists who had completed a terminal master’s program (*n* = 5) and Ph.D. students nearing completion of their degree (*n* = 3). Geologists who had not completed a Ph.D. at the time of this study performed as well as participants who had completed a Ph.D., and, as such, their data were included during analysis. All 28 geologists completed the virtual environment navigation tasks. Eighteen geologists were also administered a psychometric measure, detailed in the following, and a questionnaire to collect demographics, handedness, specific education level, area of specialty and whether and how much time they had in the field. Of the 18 geologists for whom we have data, 17 identified as white and one identified as mixed race.

For the expert comparison group, a total of 27 experienced psychologists (12 female; *M* age = 37 years, *SD* = 11.63) were recruited via email. The majority of psychologists (*n* = 19) held a Ph.D. at the time of participation in this study. Data were also collected from psychologists who had completed a terminal master’s program (*n* = 4) and Ph.D. students nearing completion of their degree (*n* = 4). All 27 psychologists completed the virtual environment navigation tasks and were also administered a psychometric measure, detailed in the following, and a questionnaire on demographics, handedness, specific education level and area of specialty; however, data for one psychologist was not recorded due to a computer crash. Of the 26 psychologists who reported on racial information, 16 psychologists identified as white, six as Asian, one as African-American and three as mixed race.

Our larger comparison group comprised 294 undergraduate students (168 female, two did not report gender) between the ages of 18 and 40 years from a large urban research university who participated in one of four studies which assessed them on Virtual Silcton performance. These data were reported previously in two manuscripts (Weisberg & Newcombe, 2016; Weisberg et al., 2014). In those studies, undergraduates who did not complete the second session of any study were excluded, but all undergraduates for whom we have Virtual Silcton data are included here. Age was not collected. One undergraduate identified as American Indian, 26 as Asian, two as Black/Hispanic, 37 as Black, six as Hispanic, 10 as White, 133 as White/non-Hispanic, six as other, four omitted this information and data were not collected for 69. Finally, we included the 77 geoscientists tested by Hegarty et al. (2010) for a comparison of self-reported SBSOD scores.

The current research received the university’s Institutional Review Board approval (Protocol number 13394: ‘Computer-Based Spatial Abilities’).

### Materials

Geologists who were recruited via email (*N* = 10) completed the study on their own personal computers. None of these participants reported any technological issues. Geologists who were recruited at conferences (*N* = 18) and all other participants completed the study on a Windows 10 64-bit computer. The computer had an Intel Core i7-4720HQ CPU @ 2.60 GHz and Nvidia GeForce GTX 960 M video card. The virtual environment (VE) was displayed on a 34.54 cm × 19.43 cm LCD monitor with a refresh rate of 60 Hz and resolution of 1920 × 1080. The VE was modeled on a real-world college campus (Schinazi et al., 2013; Weisberg et al., 2014) using Unity3D and Google Sketchup. The VE was designed to replicate the saliency and spatial location of buildings and nonbuilding objects like trees, trashcans and so forth, without replicating the exact architecture of the real-world structures (Schinazi et al., 2013).

### Virtual environment navigation paradigm (Virtual Silcton)

**Fig. 2:**
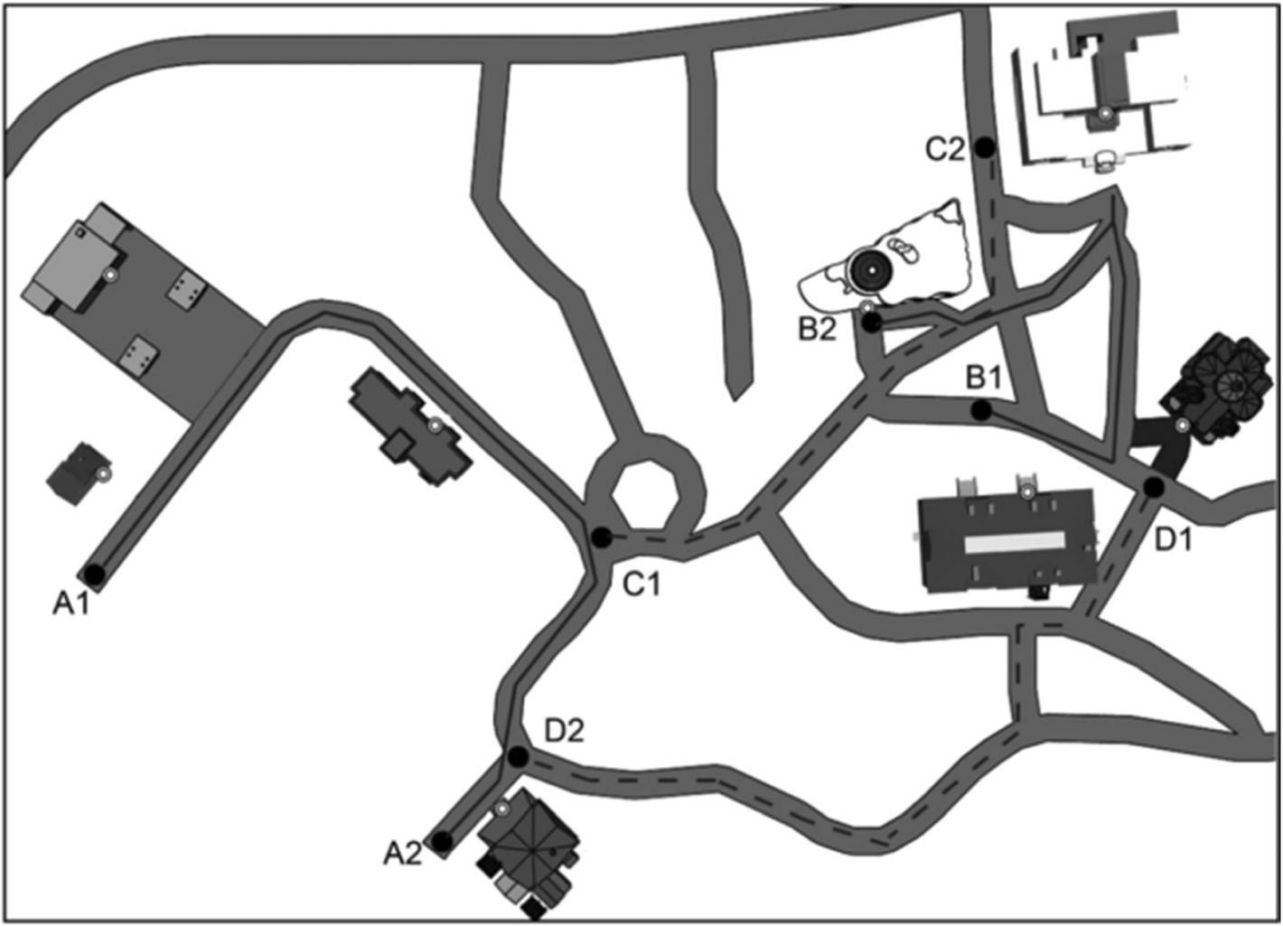
Aerial view map of Virtual Silcton showing the two main routes (solid lines A and B), the two connecting routes (dashed lines C and D) and the layout of buildings on each route. The letter–number combinations are used to indicate the start and end points along each of the main and connecting routes (i.e., participants walked from point 1 to point 2 and then back to point 1 for each of the main and connecting routes, thus traversing each route twice). Participants had to learn the names and locations of four target buildings on each of the two main routes. The presentation of the main routes were counterbalanced (A first or B first) and the presentation of the connecting routes were counterbalanced (C first or D first)

Virtual Silcton is a desktop-based virtual environment (VE) navigation paradigm. It comprises two main routes in different areas of the same VE and two connecting routes (see Fig. 2). Each main route consists of four unique target buildings for a total of eight target buildings. In the learning phase, participants were first instructed to learn the names and locations of each of the eight target buildings by virtually walking along each main route indicated by red arrows. They were told to pay attention to the front door of each building, as that was the specific spot they would be asked to point at later in the experiment. Target buildings in the VE were indicated by a blue gem hovering near the name of the target building. The two main routes were counterbalanced between participants. Participants walked from the start of each route to the end and then back to the start; thus, each route was completed twice before moving on to the next route. They were told not to veer off the path marked by red arrows, but that they could take as much time as they liked on each route. Each of the routes was surrounded by invisible walls, which kept the participant along the arrowed routes. Participants used the arrow keys on a computer keyboard to move along the virtual paths and a computer mouse to look 360^o^ around the VE. The experimenter encouraged participants to practice using the controls and to ask clarification questions before beginning the task. After learning the four target buildings on each main route, participants learned how the eight target buildings were related by walking down two connecting routes.

Before starting the two connecting routes, participants were told that these paths would “connect” or “go in between” the first two paths they had just learned. The experimenter noted that these connecting routes would not include any new buildings for participants to remember, and that instead their role was to help participants understand how the buildings related to one another. Similar to the main routes, the connecting routes were counterbalanced between participants (but always occurred after the main routes were learned). Participants were reminded to stay on the route marked by red arrows and the invisible walls along the connecting routes prevented participants from veering off-course.

In the testing phase, participants completed two spatial tasks—a pointing task and a model-building task—which tested the participant’s ability to create accurate and integrated representations of the virtual environment. In addition to the two spatial tasks, participants completed a cued building recognition task.

#### Pointing task

**Fig. 3:**
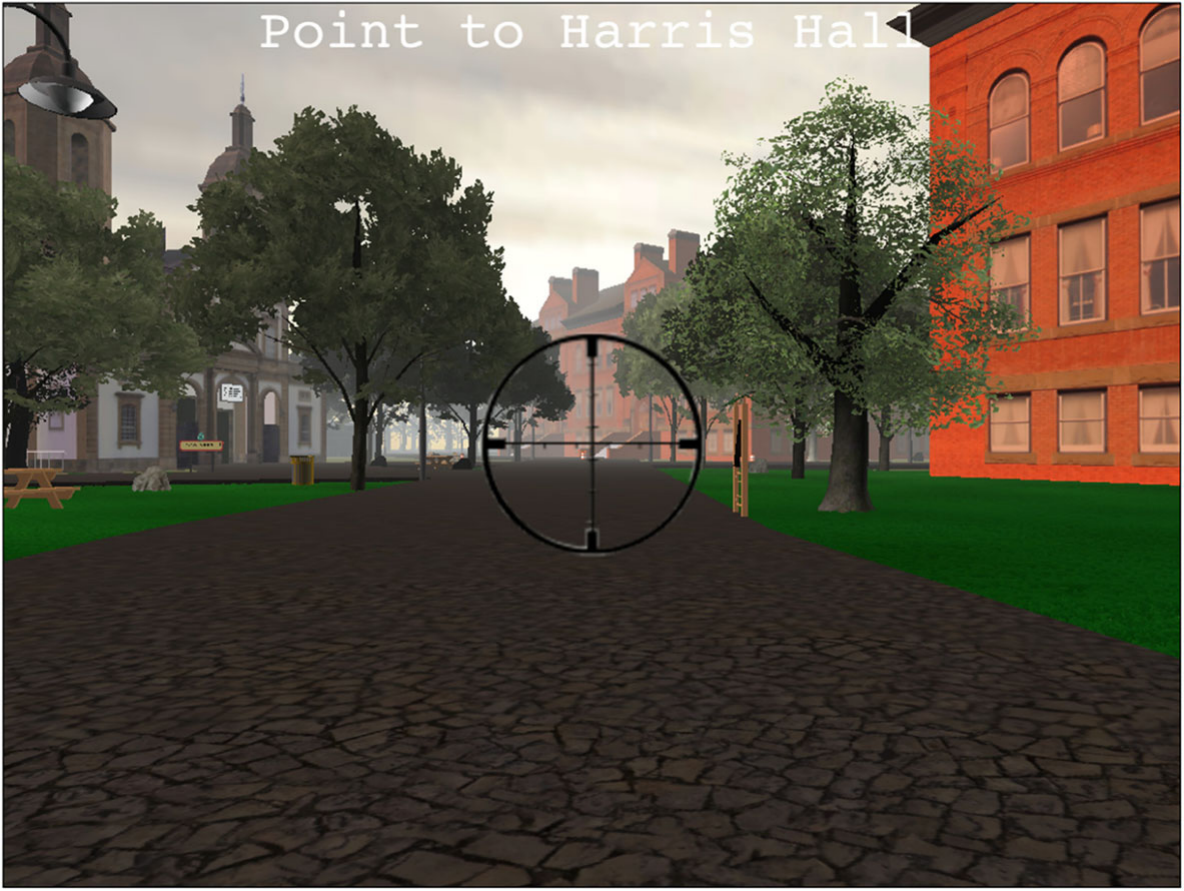
Pointing task. Participants could rotate a virtual crosshair 360^o^ along the horizontal plane to point in the direction of a target building

In the pointing task, participants were located next to one of the eight target buildings and were prompted to point in the direction of each of the other seven buildings using a virtual crosshair (see Fig. 3). Thus, three of the seven buildings would be on the same route as that of the participant in the VE and four buildings would be on the second main route. Participants pointed a virtual crosshair by rotating on the horizontal plane using the mouse in the direction of the front door of the target building and recorded their response by clicking. They were instructed to point their crosshair, specifically, at the front door of each building, and to be careful to only click once to record their answer. This process was repeated for each of the eight buildings in the VE. A pointing error score for each participant was calculated based on the absolute value of the participant’s answer minus the correct answer. If that value exceeded 180, we corrected it by subtracting the value from 360. Performance on the pointing task was subdivided into a within-route and a between-route pointing performance based on the position of the target building in relation to the participant’s pointing location in the VE. A within-route error score was calculated for trials in which the target building was on the same route as that of the participant. A between-route error score was calculated for trials in which the target building was on a different main route to that of the participant. There were a total of 24 within-route trials and 32 between-route trials.

#### Model-building task

In the model-building task, participants were told that they would construct a map of the virtual environment using a bird’s-eye view. Participants were shown an aerial view of the eight buildings and their names alongside a blank box on a computer screen. Participants had to drag and drop the miniature models of the eight buildings into the blank box at spatial locations relative to each other in order to recreate a map-like representation of the virtual environment in the blank box. A bidimensional regression analysis (Friedman & Kohler, 2003; Tobler, 1994) was used to calculate the *R*^2^ value for each participant. The *R*^2^ value corrects for rotational, translational and scale differences between the participant map and the actual map, and indicates the remaining proportion of variance in the participant’s map accounted for by the actual map. It can be interpreted as configurational accuracy.

### Psychometric measures

The Santa Barbara Sense of Direction Scale (SBSOD; Hegarty, Richardson, Montello, Lovelace, & Subbiah, 2002**)** is a self-report measure of one’s “sense of direction” or the ability to orient oneself within an environment. The measure consists of 15 items using a 7-point Likert scale with statements such as “I very easily get lost in a new city.”

### Procedure

After consenting to participate, participants completed a short demographic form to collect information such as age, education level and area of expertise. Participants then completed a computerized version of the SBSOD, followed by the virtual environment navigation paradigm. Participants were instructed to learn the names and locations of the eight target buildings as they explored the two main routes and two connecting routes in Virtual Silcton. Participants were then asked to complete the pointing and model-building tasks. Finally, participants were debriefed and thanked for their participation. The entire study, from start to finish, took approximately 45 min–1 h to complete.

## Results

We first evaluated whether self-reported navigation skill as measured by the SBSOD differed between psychologists and geologists in the current sample. We also included the larger sample of 77 geoscientists (41 female; *M* age = 34.98 years, *SD* = 11.96) tested by Hegarty et al. (2010) and undergraduate students tested by Weisberg et al. (2014, 2016). As hypothesized, one-way ANOVA revealed significant differences across the four groups, *F*(3,417) = 28.88, *p* < 0.001. A post-hoc test revealed no significant differences in SBSOD scores between the geologists in the current study (*M* = 5.12, *SD* = 1.06) and geoscientists in the Hegarty et al. survey (*M* = 5.50, *SD* = 0.86) (*p* = 0.18, *d* = 0.40, Bayes factor (*B*) = 1.04), suggesting that the current sample is not likely to be different from the discipline at large.

There was a significant difference in scores between the psychologists (*M* = 4.65, *SD* = 1.18) and the geoscientists (*p* < 0.001, *d* = 0.82), but not between the psychologists and the geologists (*p* = 0.08, *d* = 0.42). The psychologists did not differ in scores from the undergraduates (*M* = 4.35, *SD* = 0.99, *p* = 0.14, *d* = 0.28), but the undergraduates’ scores were significantly lower than the geologists (*p* < 0.001, *d* = 0.74) and the geoscientists (*p* < 0.001, *d* = 1.24). Thus, we largely confirmed the self-reported findings of Hegarty et al. (2010). However, do these differences in self-reports of environmental spatial abilities extend to an objective measure of navigation skill?

### Navigation performance in the virtual environment

There were significant group differences in the within-route pointing task, *F*(2,345) = 4.10, *p* = 0.02, *η*^2^ = 0.02. Geologists (*M* = 16.9, *SD* = 10.0) significantly outperformed undergraduate students (*M* = 23.7, *SD* = 11.9) (*p* = 0.004, *d* = 0.62). Psychologists and undergraduate students did not differ (*p* = 0.82, *d* = 0.05). However, there were no significant differences between geologists and psychologists (*M* = 23.1, *SD* = 14.5) (*p* = 0.06, *d* = 0.50), although the *d* value is large.

There were also significant group differences in the between-route pointing task, *F*(2,345) = 6.40, *p* = 0.002. Geologists (*M* = 35.7, *SD* = 19.3) significantly outperformed both psychologists (*M* = 46.88, *SD* = 17.22) (*p* = 0.005, *d* = 0.61) and undergraduate students (*M* = 45.7, *SD* = 13.7) (*p* = 0.001, *d* = 0.60). Psychologists and undergraduate students did not differ (*d* = 0.07).

Finally, there were significant group differences on the model-building task, *F*(2,344) = 11.55, *p* < 0.001. Geologists (*M* = 0.72, *SD* = 0.22) significantly outperformed both psychologists (*M* = 0.50, *SD* = 0.29) (*p* = 0.002, *d* = 0.85) and undergraduate students (*M* = 0.47, *SD* = 0.26) (*p* < 0.001, *d* = 1.04). Psychologists and undergraduate students did not differ (*d* = 0.11).

### Types of navigators

Previously we found that navigators clustered along two dimensions—performance on within-route and between-route pointing—into three groups (Weisberg & Newcombe, 2016; Weisberg et al., 2014). One group performed well on both tasks (integrators), and another performed poorly on both (imprecise navigators). A third group performed well on within-route pointing but poorly on between-route pointing (non-integrators). The ratio of navigators falling into each of these groups was approximately 1:2:1 (integrators:non-integrators:imprecise navigators).

**Fig. 4:**
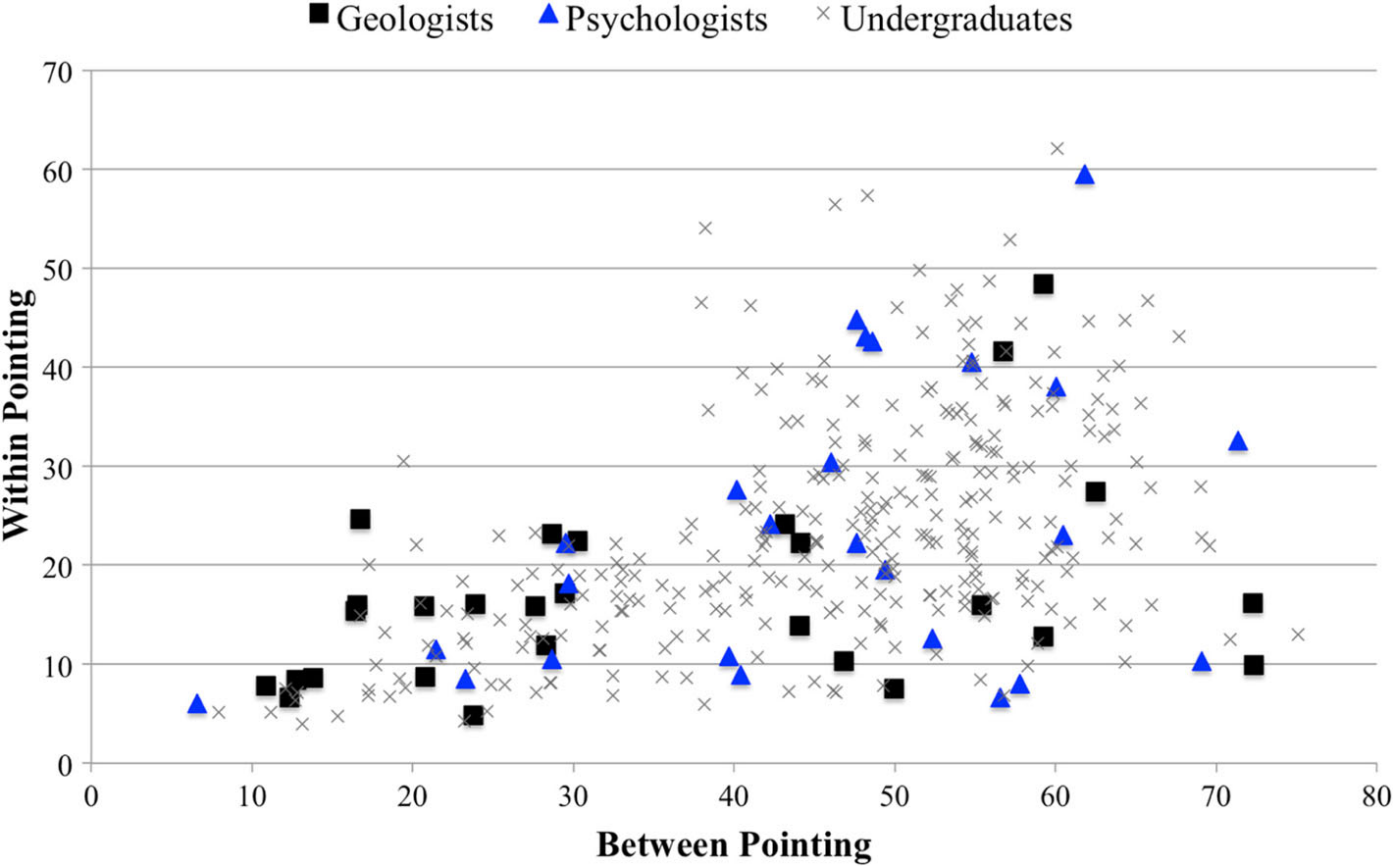
Scatterplot of performance on between-route and within-route pointing trials grouped by geologists and non-STEM undergraduates. Quadrants are based on cluster membership cutoff values—good between/good within (integrators), good between/bad within (non-integrators) and bad between/bad within (imprecise navigators)—established in previous studies using Virtual Silcton (Weisberg & Newcombe, 2016; Weisberg et al., 2014). STEM science, technology, engineering and mathematics

Figure 4 displays the scatter plot resulting from plotting the performance on between-route trials on the *x* axis and the within-route pointing performance on the *y* axis. As is visually apparent, more geologists are in the lower left of the graph, proportionally, than psychologists and undergraduate students, relative to the lower-right and upper-right quadrants. To address this analytically, due to the small sample size of geologists, we used the cutoff values from the undergraduate data to determine the number of participants in each navigator group (integrator:non-integrator:imprecise navigator). This resulted in a significant cluster difference between geologists (16:10:2), psychologists (9:9:8) and undergraduate students (84:131:79), χ^2^ (4, *N* = 348) = 11.88, *p* = 0.02, Cramer’s *V* = 0.13. A post-hoc test—using adjusted residuals and a Bonferroni correction for multiple comparisons between nine cells (three groups × three types of navigators)—showed that the number of geologists categorized as integrators (*p* = 0.0019) was significantly higher than integrators among psychologists and undergraduates, respectively. No other cells were significantly different from each other.

## Discussion

Study 1 broadens our understanding of the relation between spatial skills and STEM to an underinvestigated kind of large-scale spatial reasoning, namely the extrinsic–dynamic spatial processes related to navigation. Our results align with the findings of Hegarty et al. (2010) in that geologists report significantly higher levels of navigation skills as compared to psychologists who have completed comparable years of study and experience in the field and to non-STEM undergraduate students. Using a virtual navigation paradigm, we found that geologists indeed exhibit higher navigational competence—as measured by pointing and model-building tasks—as compared to psychologists and non-STEM students. Thus, geologists not only report higher levels of navigation skills but also demonstrate superior navigation performance than their non-STEM counterparts, comparable in years of professional training. The lack of significant differences in navigation performance between psychologists and non-STEM students further lends support to the hypothesis that additional years of non-STEM education alone do not suffice to improve large-scale spatial skills substantially. Thus, our results provide evidence for the link between large-scale navigation skills and training in the GEO fields.

Such a link is not entirely surprising given the integral nature of navigation in data collection for geology. Solid earth data are often collected over a large field area where one data collection location is not visible from another. For a field-based project, most geology Ph.D. students would collect data over an area greater than 100 km^2^ for their theses. Thus, GEO experts must coordinate multiple extrinsic relationships—between the location of the self and the map to record where data were collected, among data points on the map, between the spatial relations in a rock sample and regional spatial patterns, and among locations in the field area to decide where next to collect data (Shipley & Tikoff, in press). Geologists, who are good at efficiently covering a mapping area, spending more time in the areas that provided the highest quality information for discerning among possible interpretations, tend to be more accurate in inferring the underlying geological structure (Baker, Johnson, Callahan, & Petcovic, 2016). Finally, there is the practical advantage of being a good navigator in a field that often requires working away from established paths—good navigators are more likely to return quickly and successfully to their vehicles at the end of the day.

One of the limitations of Study 1, however, is that it is impossible to hypothesize about the domain expert’s spatial competence prior to their domain training. Do GEO experts get better at large-scale and/or small-scale spatial tasks because of their training? Or do high-spatial individuals self-select to specific STEM disciplines? Or do both effects occur? To overcome this drawback as well as to delineate the role of fieldwork in improving spatial skills, we investigated novices in a related GEO discipline requiring large-scale spatial reasoning and pattern detection across space and time.

## Study 2

In Study 2, we explored the links between self-selection to a STEM field—Geographic Information Systems (GIS)—and improvement in navigation skills after extended exposure to domain knowledge from that field. GIS involves the use of an integrated toolbox of hardware and software systems and processes designed to allow an individual to store, retrieve, visualize and transform spatial data. Over the last three decades, GIS applications have extended beyond the field of geography and into various educational domains (Madsen & Rump, 2012) with the ultimate goal to enhance our ability to address planning and management problems (National Research Council, 2006). Not unlike the field of geology, GIS entails large-scale spatial reasoning and transformations, albeit through a different medium of learning. Where geology expertise often relies on fieldwork in the real world, GIS training focuses on a technology-assisted ability to store, visualize and manipulate digitized spatial information. So, does a suite of spatial visualization and analyses software at a figural scale demand high large-scale spatial thinking and does domain-specific knowledge in this GEO field translate into better spatial skills, specifically navigation skills?

Lee and Bednarz (2009) found that students enrolled in a GIS course outperformed a control group on a spatial test. In addition, GIS participants showed significant improvement in spatial thinking during the semester. However, the questions on the spatial test created to measure spatial thinking skill were closely related to the GIS course work and as such may not have been reflective of domain-general large-scale and small-scale spatial skills. Similarly, Hall-Wallace and McAuliffe (2002) found a significant positive correlation between small-scale spatial skills—measured by the surface development and cubes comparison tasks—and GIS learning. Although limited, there is a growing body of research investigating the relation between spatial thinking skills and GIS learning (e.g., Albert & Golledge, 1999; Baker & Bednarz, 2003; Britz & Webb, 2016; Kim & Bednarz, 2013). However, research so far has been limited to small-scale spatial thinking and to spatial tests closely related to the GIS curriculum.

In Study 2, we compared large-scale and small-scale spatial skills of novice GIS students with students enrolled in a nonspatial communications (COM) course at the start (T1) and end (T2) of an academic semester. As in Study 1, participants in Study 2 completed a virtual navigation paradigm in addition to mental rotation and spatial working memory tasks. Spatial and nonspatial skill at T1 was used as a baseline to examine improvement over the course of a semester. We hypothesized that: GIS students will have significantly better spatial skills at T1 as compared to COM students; GIS students will show greater improvement in spatial skills, specifically in navigation skills, from T1 to T2 compared to COM students; and mental rotation and spatial working memory may mediate the relation between academic course and spatial skills improvement.

## Method

### Participants

A total of 90 undergraduate students (55 female) agreed to participate in the current study. Participants were recruited from introductory GIS (*n* = 47; 26 female) and communication (*n* = 43; 29 female) courses at Temple University. GIS introductory courses at the university where data were collected have an average class size of 12 students, and the goal was to collect as much data as possible over a period of 2 years (two Fall and two Spring semesters). Of the 90 participants who signed up for the study, 70 participants completed both pre-test (T1) and post-test (T2) components of the study. An equal number of GIS and COM students dropped out at T2. Age was not recorded but ranged between 18 and 25 years, which was an eligibility criterion for participation. Of those participants who chose to disclose their racial and ethnic information, four participants identified as American Indian, eight as Asian, seven as Black/African American, two as more than one race, one as Native Hawaiian, 31 as White and two as other race. The current research received the university’s Institutional Review Board approval (Protocol number 23379: ‘Exploring Links between STEM Success and Spatial Skills: Undergraduate GIS Courses and a Spatial Turn of Mind’). Participants received a $15 gift card on completion of T1 and an additional $20 gift card on completion of T2.

### Materials

The study was administered on a Windows 7 64-bit computer. The computer had an Intel Core i5–6600 CPU @ 3.30 GHz and Nvidia GeForce GT 610 video card. The virtual environment (VE) was displayed on a 40 cm × 62 cm LCD monitor with a refresh rate of 60 Hz and resolution of 1680 × 1050.

### Virtual environment navigation paradigm (Virtual Silcton)

The virtual environment navigation paradigm in Study 2 was identical to that of Study 1. After exploring the VE, participants completed the pointing task followed by the model-building task. In addition to Virtual Silcton, participants completed three psychometric and self-report measures: a mental rotation test, a spatial working memory task and a verbal ability test.

### Psychometric and self-report measures

The Mental Rotation Test (MRT; Vandenberg & Kuse, 1978, adapted by Peters et al., 1995**)** consists of 20 items each made up of one target figure and four response items. Two of the four response items are identical to the target figure, but presented at varying orientations. The remaining two items are mirror images of the target figure in varying orientations. Participants were asked to identify the two response items that were identical but rotated images of the target figure. Before beginning the task, participants were given three practice trials. If they got any of the practice problems incorrect, they reviewed their answers with the experimenter and found the right one before moving on to the actual task. Participants received 2 points for each correct response and lost 2 points for each incorrect response.

The Spatial Working Memory Complex Span (Symmetry span; Unsworth, Heitz, Schrock, & Engle, 2005) was also used. For the spatial working memory (SWM) task, participants had to remember the location of one red square in a 4 × 4 matrix of otherwise white squares. They then had to judge whether a separate array of black and white squares were bilaterally symmetrical or not. After a series of between three and five items (e.g., red square, symmetry judgment, red square, symmetry judgment, etc.), participants must recall the red square locations in the correct order. Participants’ scores are calculated by summing all correctly recalled items.

The Wide Range Achievement Test, Word Reading Subtest (WRAT-4; Wilkinson & Robertson, 2006) is a measure of verbal IQ that correlates very highly with the WAIS-III and WISC-IV (Strauss, Sherman, & Spreen, 2006). The WRAT-4 Word Reading Subtest requires participants to pronounce 55 individual words. Each participant’s score is the number of words pronounced correctly out of 55.

The Philadelphia Verbal Ability Scale (PVAS; Hegarty et al., 2010) is a self-report measure of how good participants feel their verbal ability is. The measure comprises 10 items using a 7-point Likert scale (Cronbach’s α = 0.78) with statements like “I am very good at scrabble.”

The Philadelphia Spatial Ability Scale (PSAS; Hegarty et al., 2010) is a self-report measure of how well participants feel they can perform on common small-scale spatial tasks. The measure comprises 16 items using a 7-point Likert scale (Cronbach’s α = 0.77) with statements like “I enjoy putting together puzzles.”

### Procedure

All participants completed the pre test (T1) within the first 3 weeks of the semester and completed the post test (T2) during the last 3 weeks of the semester. We ensured that the number of weeks between the pre and post tests stayed approximately constant across participants. During the pre test (T1), participants signed a consent form informing them about the two-timepoint study. Participants could opt out at any point during the study. On consenting to participate, the investigator first administered the WRAT. Participants then filled out a short demographic form and completed the online version of the mental rotation task. This was followed by the virtual environment navigation paradigm. Participants were instructed to explore the two main routes and two connecting routes in Virtual Silcton, and to complete the pointing and model-building tasks. Finally, participants completed an e-prime version of the SWM measure. The entire study from start to finish took approximately 1 h per session and not more than 2.5 h for both sessions.

## Results

To evaluate our three hypotheses, we ran repeated-measures ANOVA followed by post-hoc tests to compare baseline competency and improvement over time in the navigation and mental rotation skills of GIS and COM participants. We also ran mediation models to investigate the role of mental rotation and spatial working memory in improving navigation skills.

Prior to analysis, the data were evaluated for multivariate outliers by examining leverage indices for each individual (Jaccard & Wan, 1993). No outliers were detected. Further, a Levene’s test for homogeneity of variance demonstrated that the assumption of equal variances was met (all *p >* 0.05). Our sample had missing data (approximately 22% attrition; i.e., participants who completed T1 but did not return for testing at T2). To deal with the missing data, we ran a multiple imputation analysis using SPSS v20 and followed the guidelines for multiple imputation analysis specified in Jeličić, Phelps, and Lerner (2009) (see also Rezvan, Lee, & Simpson, 2015, for a review). The MI analysis was conducted using 23 imputations so as to exceed the percentage of attrition that was found to be approximately 22% (White, Royston, & Wood, 2011). All of the following analyses were conducted using the imputed dataset and all figures/tables present in the imputed dataset.

### Baseline and improvement in navigation skills

Table 1 presents descriptive statistics for the spatial tasks and psychometric measures grouped by participant course. In order to test for baseline competency and improvement in spatial performance over time moderated by participant course, we ran repeated-measures ANOVAs followed by post-hoc *t* tests. GIS and COM participants were comparable on nonspatial verbal ability as measured by the WRAT, *t*(88) = 1.49, *p* = 0.14, *d* = 0.32, and the PVAS, *t*(88) = 0.94, *p* = 0.35, *d* = 0.20. There were also no significant differences between the groups on the PSAS, *t*(88) = 1.64, *p* = 0.10, *d* = 0.35.

**Table 1:** Descriptive statistics by course for T1 and T2

	GIS Mean (SD)		COM Mean (SD)	
	T1	T2	T1	T2
Within-route	20.27 (11.47)	15.16 (8.86)	26.04 (13.73)	23.69 (10.34)
Between-route	43.61 (11.71)	35.16 (15.13)	47.58 (16.00)	46.13 (14.44)
Model-building	0.5277 (0.29)	0.6393 (0.28)	0.4503 (0.27)	0.4487 (0.29)
MRT	34.13 (21.79)	42.53 (22.09)	22.76 (17.93)	23.46 (23.63)
SWM	28.57 (7.31)	30.90 (7.44)	22.98 (9.32)	26.57 (9.72)
WRAT	47.98 (4.32)	–	46.26 (6.48)	–
PSAS	4.90 (0.75)	–	4.64 (0.75)	–
PVAS	4.68 (0.86)	–	4.49 (1.05)	–

*COM* Communication, *GIS* Geographic Information Systems, *MRT* Mental Rotation Test, *PSAS* Philadelphia Spatial Ability Scale, *PVAS* Philadelphia Verbal Ability Scale, *SD* standard deviation, *SWM* Spatial Working Memory, *T1* pre test (start of academic semester), *T2* post test (end of academic semester), *WRAT* Wide Range Achievement Test

#### Within-route pointing error

**Fig. 5:**
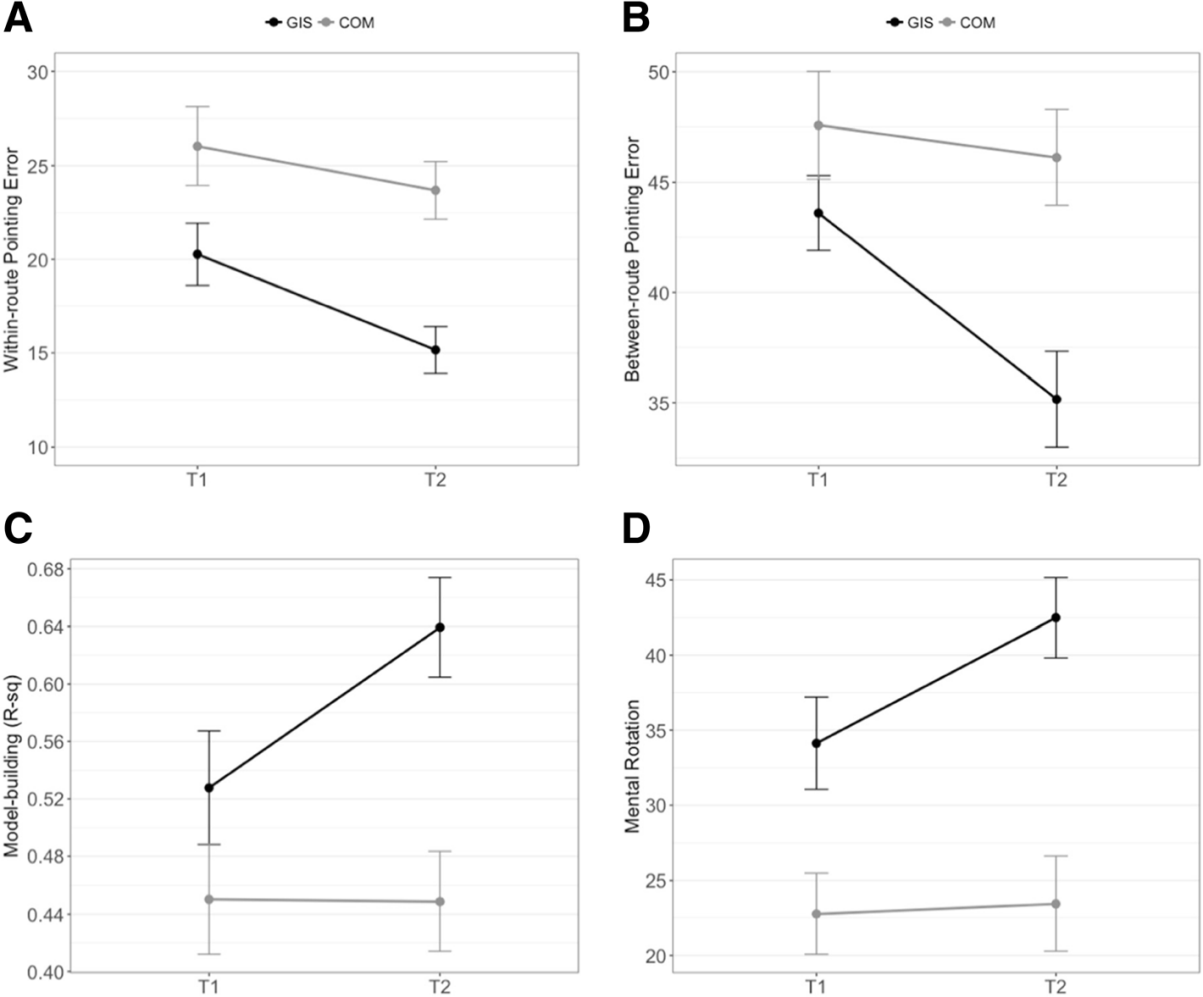
Change in large-scale navigation and small-scale mental rotation tasks, grouped by participant course. **a** Within-route pointing error. **b** Between-route pointing error. **c** Model-building (R^2^ value). **d** Mental rotation skill. Overall, GIS participants significantly outperformed COM participants and there was significant improvement in both groups from T1 to T2. In addition, GIS participants improved at a faster rate than COM participants on all measures except the within-route pointing task. Error bars reflect ±1 standard error of the mean. COM Communication, GIS Geographic Information Systems, T1 pre test (start of academic semester), T2 post test (end of academic semester). For within- and between- pointing errors, a low value (error) indicates high accuracy

There was a significant main effect of participant course, *F*(1,88) = 11.53, *p* < 0.001, partial *η*^2^ = 0.12, and time, *F*(1,88) = 13.74, *p* < 0.001, partial *η*^2^ = 0.14. However, there was no significant interaction between time and course, *F*(1,88) = 1.88, *p* = 0.17, partial *η*^2^ = 0.02 (see Fig. 5a). Thus, overall, GIS participants outperformed COM participants on the within-route pointing trials and there was significant improvement from T1 to T2 for both groups. However, there was no significant difference in the rates of improvement from T1 to T2. A *t* test revealed that at baseline GIS participants were significantly better than COM participants on the within-route pointing task, *t*(88) = 2.17, *p* = 0.03, *d* = 0.46. This task was further divided into seen and unseen within-route trials based on the intervisibility of target buildings along a route. The pattern of results is consistent with the overall within-route pointing error, with no significant differences between trials when the target was visible or not.

#### Between-route pointing error

There was a significant main effect of course, *F*(1,88) = 8.00, *p* < 0.01, partial *η*^2^ = 0.08, and time, *F*(1,88) = 12.05, *p* < 0.001, partial *η*^2^ = 0.12. More importantly, there was a significant interaction between time and course, *F*(1,88) = 6.02, *p* = 0.02, partial *η*^2^ = 0.06 (see Fig. 5b). The significant interaction between time and course is indicative of the differences in slopes (i.e., differences in rates of improvement from T1 to T2 in favor of GIS participants). At baseline, GIS participants were not significantly better than COM participants on the between-route pointing task, *t*(88) = 1.35, *p* = 0.18, *d* = 0.26.

#### Model-building (*R*^2^ value)

There was a significant main effect of participant course, *F*(1,88) = 13.08, *p* < 0.001, partial *η*^2^ = 0.13, and time, *F*(1,88) = 4.44, *p* = 0.04, partial *η*^2^ = 0.05 (see Fig. 5c). More importantly, there was a significant interaction between time and course, *F*(1,88) = 4.70, *p* = 0.03, partial *η*^2^ = 0.05 (see Fig. 5c). Similar to the between-route pointing task, there were differences in rates of improvement from T1 to T2 in favor of GIS participants. At baseline, GIS participants were not significantly better than COM participants on the model-building task, *t*(88) = 1.31, *p* = 0.19, *d* = 0.28.

#### Mental rotation skill

There was a significant main effect of participant course, *F*(1,88) = 15.47, *p* < 0.001, partial *η*^2^ = 0.15, and time, *F*(1,88) = 10.34, *p* = 0.002, partial *η*^2^ = 0.12. There was a significant interaction between time and course, *F*(1,88) = 7.42, *p* < 0.01, partial *η*^2^ = 0.08 (see Fig. 5d). Similar to the between-route and model-building performances, we found differences in rates of improvement from T1 to T2 in favor of GIS participants. At baseline, GIS participants were significantly better than COM participants on the MRT, *t*(88) = 2.69, *p* = 0.01, *d* = 0.57.

#### Spatial working memory

There was a significant main effect of participant course, *F*(1,88) = 10.49, *p* = 0.002, partial *η*^2^ = 0.12, and time, *F*(1,88) = 30.88, *p* < 0.001, partial *η*^2^ = 0.26, but no significant interaction between time and course, *F*(1,88) = 1.40, *p* = 0.24, partial *η*^2^ = 0.02. Thus, overall, GIS participants outperformed COM participants on SWM and there was significant improvement from T1 to T2 for both groups. However, there was no significant difference in the rates of improvement from T1 to T2. At baseline, GIS participants were significantly better than COM participants on the SWM task, *t*(88) = 3.18, *p* < 0.001, *d* = 0.67.

These analyses were also run using listwise deletion instead of multiple imputations. All results stayed the same except in the case of model-building performance. There was no significant main effect of time or time × course interaction. However, listwise deletion is a less optimal strategy for dealing with missing data in a longitudinal design and can reduce statistical power with small sample sizes (Acock, 2005). Hence, we used results of the multiple imputations to interpret our findings. The presented analyses were also run controlling for verbal ability as measured by the WRAT. There were no changes in our findings; we do not present these additional analyses for the sake of brevity.

### Types of navigators

**Fig. 6:**
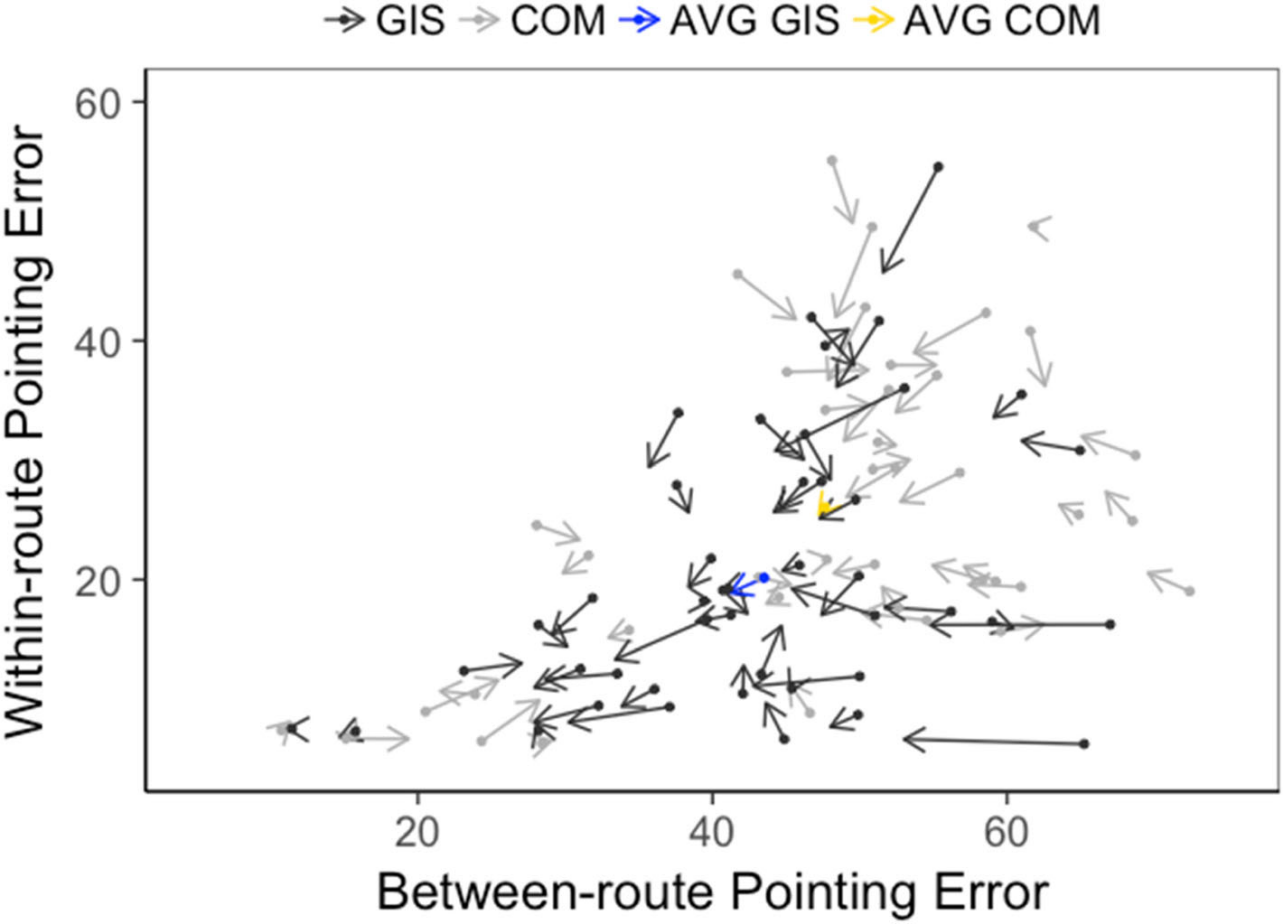
Quiver plot of performance on between-route and within-route pointing trials at T1 and T2 grouped by participant course. Arrows depict the magnitude (scaled) and direction of change in performance from T1 to T2. AVG average, COM Communication, GIS Geographic Information Systems, T1 pre test (start of academic semester), T2 post test (end of academic semester)

Figure 6 is a quiver (velocity) plot of participant performance along two dimensions: performance on within-route and between-route pointing. The arrow length and direction represent the scaled magnitude of change and the direction of change in pointing performance from T1 to T2 (down and to the left represent improvement). As is visually apparent, on average GIS participants (blue arrow) showed more improvement than COM participants (yellow arrow). To test this pattern statistically, we ran a constrained cluster analysis (number of clusters = 3), similar to that conducted in Study 1 and in previous studies (Weisberg & Newcombe, 2016; Weisberg et al., 2014). At T1, there was no significant relation between cluster-membership (integrators:non-integrators:imprecise navigators) between GIS (11:26:10) and COM (10:15:18) participants, *χ*^2^(2, *N* = 90) = 5.12, *p* = 0.08, Cramer’s *V* = 0.24. At T2, a chi-square analysis found a significant difference in cluster membership between GIS (15:25:7) and COM (7:19:17) participants, *χ*^2^(2, *N* = 90) = 7.73, *p* = 0.02, Cramer’s *V* = 0.29. GIS participants were more likely to be integrators and COM participants were more likely to be imprecise navigators. Overall, 60 participants (GIS = 33, COM = 27) recorded no change in cluster membership from T1 to T2, 17 participants (GIS = 10, COM = 7) demonstrated a positive change (i.e., they moved into a better navigator category from T1 to T2) and 13 participants (GIS = 4, COM = 9) recorded a negative change (i.e., they moved into a lower navigator category from T1 to T2). However, there was no significant difference in change in cluster membership between the two groups, *χ*^2^(2, *N* = 90) = 2.88, *p* = 0.24, Cramer’s *V* = 0.18.

### Role of mental rotation and spatial working memory in navigation skills

We found significant differences in mental rotation and spatial working memory between GIS and COM participants, which were parallel to differences on the navigation tasks. Hence, mental rotation and spatial working memory at T1 were assessed as potential mediators influencing the relation between course and navigation performance at T2, controlling for baseline performance at T1. However, neither MRT nor SWM were found to mediate the relation between course and pointing performance at T2, controlling for baseline pointing performance. The strength of the direct effect of course on within-route pointing was *b* = 5.72, *t*(87) = 3.74, *p* < 0.001, and the bootstrapped indirect effect of course on within-route pointing was *b* = 0.06 (standard error (*SE*) = 0.34, [−0.60, 0.84]) for MRT as a potential mediator and *b* = 0.20 (*SE* = 0.48, [− 0.71, 1.26]) for SWM as a potential mediator. The strength of the direct effect of course on between-route pointing was *b* = 8.69, *t*(87) = 3.33, *p* = 0.001, and the bootstrapped indirect effect of course on between-route pointing was *b* = 0.73 (*SE* = 0.82, [− 0.50, 2.63]) for MRT as a potential mediator and *b* = 0.12 (*SE* = 0.88, [− 1.48, 2.05]) for SWM as a potential mediator. Similarly, MRT and SWM did not significantly mediate the relation between course and model-building performance at T2, controlling for baseline model-building performance. The strength of the direct effect of course on model-building was *b* = − 0.16, *t*(87) = − 3.62, *p* < 0.001, and the bootstrapped indirect effect of course on model-building was *b* = − 0.02 (*SE* = 0.01, [− 0.05, 0.001]) for MRT as a potential mediator and *b* = − 0.02 (*SE* = 0.02, [− 0.06, 0.004]) for SWM as a potential mediator. Thus, the differences in navigation performance are attributable to improvements on navigation-specific processes, and not small-scale spatial skill or spatial working memory.

### Sex differences in spatial, nonspatial and psychometric measures

Although the current study was not designed specifically to examine sex differences, we ran repeated-measures analyses to examine whether improvement in spatial, nonspatial and psychometric measures were moderated by sex. Table 2 presents descriptive statistics for spatial, nonspatial and psychometric performance at T1 and T2, grouped by participant sex. For within-route pointing performance, there were no significant effects of participant sex, *F*(1,87) =2.78, *p* = 0.10, partial *η*^2^ = 0.03, time, *F*(1,87) = 3.17, *p* = 0.08, partial *η*^2^ = 0.04, or interaction between time and sex, *F*(1,87) = 0.07, *p* = 0.81, partial *η*^2^ = 0.00. Similarly, for between-route pointing performance, there were no significant effects of participant sex, *F*(1,87) = 3.83, *p* = 0.06, partial *η*^2^ = 0.04, time, *F*(1,87) = 0.58, *p* = 0.46, partial *η*^2^ = 0.01, or interaction between time and sex, *F*(1,87) = 0.36, *p* = 0.58, partial *η*^2^ = 0.00. For model-building performance, there were no significant effects of participant sex, *F*(1,87) = 2.46, *p* = 0.16, partial *η*^2^ = 0.03, time, *F*(1,87) = 1.18, *p* = 0.39, partial *η*^2^ = 0.01, or interaction between time and sex, *F*(1,87) = 0.77, *p* = 0.51, partial *η*^2^ = 0.01. In addition, we checked for sex differences in navigator type using a chi-square test of independence. There was no significant relation between participant sex and cluster membership at T1, *χ*^2^(4, *N* = 90) = 8.07, *p* = 0.09, or at T2, *χ*^2^(4, *N* = 90) = 7.15, *p* = 0.13. Thus, males and females were equally likely to be represented in each of the navigator clusters at T1 and T2.

**Table 2:** Descriptive statistics by participant sex for T1 and T2

	Male Mean (SD)		Female Mean (SD)	
	T1	T2	T1	T2
Within-route	20.16 (11.83)	16.36 (9.91)	24.85 (13.25)	21.06 (10.41)
Between-route	42.25 (15.24)	36.17 (15.71)	47.58 (12.84)	43.09 (15.34)
Model-building	0.5582 (0.2780)	0.5855 (0.3195)	0.4478 (0.2774)	0.5246 (0.2911)
MRT	35.89 (22.49)	38.47 (26.40)	24.12 (18.30)	30.20 (23.77)
SWM	27.08 (7.93)	30.38 (8.84)	25.15 (9.23)	27.84 (9.11)
WRAT	48.29 (4.97)	–	46.44 (5.73)	–
PSAS	4.74 (0.76)	–	4.80 (0.76)	–
PVAS	4.66 (0.92)	–	4.55 (0.98)	–

*MRT* Mental Rotation Test, *PSAS* Philadelphia Spatial Ability Scale, *PVAS* Philadelphia Verbal Ability Scale, *SD* standard deviation, *SWM* Spatial Working Memory, *T1* pre test (start of academic semester), *T2* post test (end of academic semester), *WRAT* Wide Range Achievement Test

However, for mental rotation skill, there was a significant main effect of participant sex, *F*(1,87) = 4.55, *p* < 0.05, partial *η*^2^ = 0.05, in favor of male participants. There was no significant main effect of time, *F*(1,87) = 0.53, *p* = 0.63, partial *η*^2^ = 0.01, and no interaction between time and participant sex, *F*(1,87) = 1.37, *p* = 0.39, partial *η*^2^ = 0.02. Thus, male participants outperformed female participants on the MRT irrespective of time. However, there were no significant differences in rates of improvement in males and females from T1 to T2. For SWM, there was no significant effect of participant sex, *F*(1,87) =1.22, *p* = 0.30, partial *η*^2^ = 0.01, time, *F*(1,87) = 0.73, *p* = 0.50, partial *η*^2^ = 0.01, or the interaction between time and sex, *F*(1,87) = 0.77, *p* = 0.55, partial *η*^2^ = 0.01.

Finally, we examined the interaction of all three variables: time, course and participant sex. For the within-route pointing task, there was a significant interaction, *F*(1,85) = 0.4.59, *p* = 0.04, partial *η*^2^ = 0.05. There was no significant interaction for the between-route pointing, model-building, MRT and spatial working memory tasks.

## Discussion

Study 2 addresses the bidirectional relation between GEO training and spatial thinking skills. We had three main hypotheses in Study 2. First, we hypothesized that GIS students will have significantly better spatial skills at baseline as compared to COM students. Our results indicate that some (but not all) baseline spatial skills of GIS students are better than those of COM participants. Thus, high-spatial individuals may be self-selecting to the high-spatial GIS discipline. However, differences in spatial skills were limited to the large-scale within-route pointing task and the small-scale mental rotation task. At baseline, GIS participants are not significantly better than COM participants at between-route pointing, which involves integrating the different routes in the VE, or at creating a map of the environment in the model-building task. One explanation for this could be that our sample size for the two groups was too small to detect significant differences for small to medium effect sizes for these variables (*d* < 0.3, Cramer’s *V* = 0.24). However, it is possible that students enrolling in the high-spatial GIS course may have some relevant spatial skills but that this advantage is non-existent or weak for exactly those kinds of abilities supported by thinking about spatial distributions and integrating them.

Second, we hypothesized that GIS students will show greater improvement in spatial skills, specifically in navigation skills from T1 to T2 as compared to COM students. As hypothesized, GIS students recorded a significantly faster rate of improvement from T1 to T2 in the between-route pointing and model-building tasks compared to COM students. In addition, GIS students showed a significantly faster rate of improvement in small-scale mental rotation skills as compared to COM students. Interestingly, spatial working memory improved for both GIS and COM students from T1 to T2 but there were no significant differences in the rates of improvement. These gains may reflect retesting effects. Taken together, our findings not only suggest the predictive role of spatial skills in self-selection to the high-spatial GIS courses but support GIS as a potential tool for improving spatial skills. It is important to note here that results of the listwise deletion approach to missing data suggest that model-building performance should be interpreted with some caution.

Finally, we hypothesized that mental rotation and spatial working memory might mediate the relation between academic course and improvement in spatial skills. However, our data did not support those ideas; baseline small-scale mental rotation skills did not mediate the relation between academic course and improvement in large-scale navigational proficiency. This finding supports the dissociation between small-scale object-based and large-scale environmental space transformations (Hegarty, Montello, Richardson, Ishikawa, & Lovelace, 2006; Hegarty & Waller, 2004; Newcombe & Shipley, 2015). Perspective-taking skills seem to play a more important role than mental rotation skills in predicting navigational performance in the VE (Nazareth, Weisberg, Margulis, & Newcombe, in press; Schinazi et al., 2013) and should be investigated in research on GIS in the future. It is more surprising that baseline spatial working memory was not found to mediate the relation between academic course and improvement in large-scale navigational proficiency, because previous research has found both verbal and spatial working memories to correlate with navigation performance (Weisberg & Newcombe, 2016).

Why did GIS seem to improve mental rotation? Arguably, GIS technology engages small-scale spatial manipulations on a computer screen, which would explain improvements in small-scale mental rotation skill over the course of a semester. For example, an introductory GIS course may require a student to solve a social or management issue by creating a graphical representation, using computer software (e.g., create a map using geographic information) and analyzing spatial patterns. In contrast, the introductory communication course for the control group may involve discussions on, but not graphical visualizations of, social and strategic communication issues.

What about the GIS curriculum aids in the development of large-scale navigation skills? One explanation is that when GIS tools are used appropriately in the classroom, the technology improves the quality of learning by immersing a student in spatial analysis and making all geographic assumptions explicit through graphical visualizations (Meyer, Butterick, Olkin, & Zack, 1999). Interactive pattern learning coupled with the visual component of GIS facilitates the understanding of the underlying geographic and spatial principles, and consequently can help in the development of spatial reasoning skills (Goodchild, 1993). In a way, GIS tools reduce the ambiguity associated with abstractions in scale, projections, geometry and topology (Bednarz & Ludwig, 1997; Self, Gopal, Golledge, & Fenstermaker, 1992). Even introductory GIS courses—like those in the current study—include large components of extrinsic–dynamic types of spatial relations and application and require students to develop GIS-based solutions to geographic (large-scale) modeling tasks. A focus on mapping principles, map overlays and cartography may further help develop perspective-taking skills, which consequently improves large-scale navigation proficiency. Of course, variations in the content and style of teaching GIS software at the university level could greatly influence improvement in large-scale navigation.

## Conclusion

Spatial skills appear to be at the core of several scientific disciplines. However, there may be differences in the amount and type of spatial demands in the STEM fields. For example, physicists and geographers study phenomena that occur at different scales. There is growing evidence for dissociations between small-scale object-based spatial skills like mental rotation and large-scale perspective-taking and navigation skills through behavioral (Hegarty et al., 2010) and functional magnetic resonance imaging (Lambrey, Doeller, Berthoz, & Burgess, 2012) findings, allowing us to identify gaps in the literature linking spatial thinking to enrollment and success in the different STEM fields. One such gap is the study of large-scale navigation skills and its relation to training and expertise in the GEO fields. Existing literature focuses almost exclusively on small-scale spatial skills and therefore little is known about large-scale spatial skills like navigation, which may be particularly important for the GEO fields. The current study provides evidence for the link between large-scale navigational competence and geology training. In Study 1 we found that geologists not only report higher self-ratings but also demonstrate higher navigational competence in a VE than non-STEM undergraduates.

Spatial skills are malleable, and gains through spatial training are durable and transfer to other skills (Uttal et al., 2013). Thus, early improvement in large-scale and small-scale spatial skills may be one route toward increasing the STEM workforce overall, and addressing a potential factor responsible for the underrepresentation of women in STEM. However, we lack a sustainable spatial training model that can be integrated into classrooms with minimum disruption in existing school and university curricula; achieving this goal requires the assessment of spatial training tools and interventions that impact the relation between STEM and spatial skills. GIS software and courses present a viable spatial training tool that can be integrated into existing school and university curricula. The effective use of GIS to promote spatial thinking depends on our ability to understand the technology, its benefits and shortcomings and its relation to specific spatial skills. Although the relation between the field of geography and the development of a “spatial turn of mind” has received some attention (e.g., Albert & Golledge, 1999; Goodchild & Janelle, 2010; Oldakowski, 2001), particularly with regard to cartography or map-reading, there is a lack of research examining how the use of GIS tools may enhance spatial thinking skills (Britz & Webb, 2016). From a cognitive perspective, the lack of systematic empirical research examining the effectiveness of GIS training in improving spatial thinking makes it difficult to identify *how* spatial skills are impacted by new spatial visualization software. We are already beginning to see the benefits of geospatial curriculum at the school level on small-scale spatial thinking (Jant, Uttal, & Kolvoord, 2014). The current article extends the literature on the benefits of GIS training to large-scale navigational skills at the university level. In Study 2, we found that novice GIS students show higher baseline mental rotation skills and, to some extent, navigational skills. However, over the course of an academic semester, GIS students improve at a faster rate than non-STEM undergraduates in both large-scale and small-scale spatial skills.

In conclusion, the current study broadens our understanding of the relation between spatial skills and STEM fields to a hitherto underinvestigated type of spatial reasoning—navigation skills. Logically, large-scale spatial skills involved in navigation should be related to STEM fields like the GEO disciplines that involve encoding and transformation of geographical and environmental space, and the current study empirically supports the bidirectional nature of this linkage.

### Limitations

In Study 1, we were limited by the amount of testing time available with expert geologists and psychologists. As a result, we were unable to administer many small-scale and large-scale assessments. Geologists may not only be better on large-scale navigation skills but may also have superior mental rotation and perspective-taking skills, which mediate the relation between discipline and navigation performance. In the absence of these data, we were unable to test more complex statistical models of difference in spatial skills between the experimental and comparison groups. Secondly, the convenience afforded by a virtual navigation paradigm comes at the cost of important proprioceptive and vestibular cues and a limited field of view (FOV), which are important for navigation (Maguire, Burgess & O’Keefe, 1999; Richardson, Montello and Hegarty, 1999). Arguably, GIS students may simply have more experience using virtual interfaces, and in the absence of this advantage may not demonstrate better navigation skills as compared to communication students in a real-world environment. Finally, we only used one measure of spatial working memory (i.e., Symmetry span) and hence findings pertaining to working memory should be interpreted with caution.

## Abbreviations

COM: Communication
FOV: Field of view
GEO: Geography and Geoscience
GIS: Geographic Information Systems
MRT: Mental Rotation Test
PSAS: Philadelphia Spatial Ability Scale
PVAS: Philadelphia Verbal Ability Scale
SBSOD: Santa Barbara Sense of Direction Scale
STEM: Science, technology, engineering and mathematics
SWM: Spatial working memory
T1: Pre test (start of academic semester)
T2: Post test (end of academic semester)
VE: Virtual environment
WRAT: Wide Range Achievement Test

## References

[CR1] Acock AC (2005). Working with missing values. Journal of Marriage and Family.

[CR2] Aguirre GK, D’Esposito M (1999). Topographical disorientation: a synthesis and taxonomy. Brain.

[CR3] Albert WS, Golledge RG (1999). The use of spatial cognitive abilities in geographical information systems: the map overlay operation. Transactions in GIS.

[CR4] Baker KM, Johnson AC, Callahan CN, Petcovic HL (2016). Use of cartographic images by expert and novice field geologists in planning fieldwork routes. Cartography and Geographic Information Science.

[CR5] Baker TR, Bednarz SW (2003). Lessons learned from reviewing research in GIS. Journal of Geography.

[CR6] Bednarz SW, Ludwig G (1997). Ten things higher education needs to know about GIS in primary and secondary education. Transactions in GIS.

[CR7] Blacker KJ, Weisberg SM, Newcombe NS, Courtney SM (2017). Keeping track of where we are: spatial working memory in navigation. Visual Cognition.

[CR8] Britz HW, Webb P (2016). The effect of an intervention using GIS-generated geo-spatial data on the promotion of spatial cognition and spatial perspective taking in grade 11 learners. South African Geographical Journal.

[CR9] Carroll JB (1993). Human cognitive abilities: A survey of factor-analytic studies.

[CR10] Chatterjee A (2008). The neural organization of spatial thought and language. Seminars in Speech and Language.

[CR11] Choi Jean, McKillop Erin, Ward Micheal, L’Hirondelle Natasha (2006). Sex-Specific Relationships Between Route-Learning Strategies and Abilities in a Large-Scale Environment. Environment and Behavior.

[CR12] Eliot J (1987). Models of psychological space.

[CR13] Friedman A, Kohler B (2003). Bidimensional regression: assessing the configural similarity and accuracy of cognitive maps and other two-dimensional data sets. Psychological Methods.

[CR14] Galati A, Weisberg SM, Newcombe NS, Avraamides MN (2017). When gestures show us the way: co-thought gestures selectively facilitate navigation and spatial memory. Spatial Cognition & Computation.

[CR15] Goodchild MF (1993). Ten years ahead: Dobson’s automated geography in 1993. The Professional Geographer.

[CR16] Goodchild MF, Janelle DG (2010). Toward critical spatial thinking in the social sciences and humanities. GeoJournal.

[CR17] Hall-Wallace MK, McAuliffe CM (2002). Design, implementation, and evaluation of GIS-based learning materials in an introductory geosciences. Journal of Geoscience Education.

[CR18] Hambrick DZ, Libarkin JC, Petcovic HL, Baker KM, Elkins J, Callahan C, Ladue ND (2012). A test of the circumvention-of-limits hypothesis in geological bedrock mapping. Journal of Experimental Psychology: General.

[CR19] Hegarty M, Crookes RD, Dara-Abrams D, Shipley TF, Hölscher C, Shipley TF, Belardinelli MO, Bateman JA, Newcombe NS (2010). Do all science disciplines rely on spatial abilities? Preliminary evidence from self-report questionnaires. Spatial cognition VII. LNAI 6222.

[CR20] Hegarty M, Montello DR, Richardson AE, Ishikawa T, Lovelace K (2006). Spatial abilities at different scales: individual differences in aptitude-test performance and spatial-layout learning. Intelligence.

[CR21] Hegarty M, Richardson AE, Montello DR, Lovelace K, Subbiah I (2002). Development of a self-report measure of environmental spatial ability. Intelligence.

[CR22] Hegarty M, Waller D (2004). A dissociation between mental rotation and perspective-taking spatial abilities. Intelligence.

[CR23] Heth C.Donald, Cornell Edward H., Alberts Denise M. (1997). DIFFERENTIAL USE OF LANDMARKS BY 8- AND 12-YEAR-OLD CHILDREN DURING ROUTE REVERSAL NAVIGATION. Journal of Environmental Psychology.

[CR24] Hölscher Christoph, Tenbrink Thora, Wiener Jan M. (2011). Would you follow your own route description? Cognitive strategies in urban route planning. Cognition.

[CR25] Ishikawa T, Montello DR (2006). Spatial knowledge acquisition from direct experience in the environment: individual differences in the development of metric knowledge and the integration of separately learned places. Cognitive Psychology.

[CR26] Jaccard, J., & Wan, C. K. (1993). Statistical analysis of temporal data with many observations: Issues for behavioral medicine data. Annals of BehavioralMedicine, 15(1), 41–50. 10.1093/abm/15.1.41

[CR27] Jant EA, Uttal DH, Kolvoord R (2014). Spatially enriched curriculum improves students’ critical thinking and spatial reasoning. Spatial Cognition 2014: Poster Presentations.

[CR28] Jeličić H, Phelps E, Lerner RM (2009). Use of missing data methods in longitudinal studies: the persistence of bad practices in developmental psychology. Developmental Psychology.

[CR29] Kali Y, Orion N (1996). Spatial abilities of high-school students in the perception of geologic structures. Journal of Research in Science Teaching.

[CR30] Kastens KA, Agrawal S, Liben LS (2008). Research in science education: the role of gestures in geoscience teaching and learning. Journal of Geoscience Education.

[CR31] Kastens Kim A., Manduca Cathryn A., Cervato Cinzia, Frodeman Robert, Goodwin Charles, Liben Lynn S., Mogk David W., Spangler Timothy C., Stillings Neil A., Titus Sarah (2009). How Geoscientists Think and Learn. Eos, Transactions American Geophysical Union.

[CR32] Kell HJ, Lubinski D, Benbow CP, Steiger JH (2013). Creativity and technical innovation: spatial ability’s unique role. Psychological Science.

[CR33] Kim M, Bednarz R (2013). Effects of a GIS Course on Self-Assessment of Spatial Habits of Mind (SHOM). Journal of Geography.

[CR34] Lambrey S, Doeller C, Berthoz A, Burgess N (2012). Imagining being somewhere else: neural basis of changing perspective in space. Cerebral Cortex.

[CR35] Lee J, Bednarz R (2009). Effect of GIS learning on spatial thinking. Journal of Geography in Higher Education.

[CR36] Linn MC, Petersen AC (1985). Emergence and characterization of sex differences in spatial ability: a meta-analysis. Child Development.

[CR37] Madsen LM, Rump C (2012). Considerations of how to study learning processes when students use GIS as an instrument for developing spatial thinking skills. Journal of Geography in Higher Education.

[CR38] Maguire Eleanor A, Burgess Neil, O’Keefe John (1999). Human spatial navigation: cognitive maps, sexual dimorphism, and neural substrates. Current Opinion in Neurobiology.

[CR39] Meyer JW, Butterick J, Olkin M, Zack G (1999). GIS in the K-12 curriculum: a cautionary note. The Professional Geographer.

[CR40] Montello DR, Frank AU, Campari I (1993). Scale and multiple psychologies of space. Spatial information theory: A theoretical basis for GIS.

[CR41] Morris RG, Parslow DM, Allen GL, Haun D (2004). Neurocognitive components of spatial memory. Remembering where: Advances in understanding spatial memory.

[CR42] National Research Council (2006). Learning to think spatially.

[CR43] Nazareth Alina, Weisberg Steven M., Margulis Katherine, Newcombe Nora S. (2018). Charting the development of cognitive mapping. Journal of Experimental Child Psychology.

[CR44] Newcombe, N. S. (2018). Three kinds of spatial cognition. In J. Wixted (Ed.), *Stevens' handbook of experimental psychology and cognitive neuroscience* (pp. 1-31). Wiley

[CR45] Newcombe NS, Shipley TF, Gero JS (2015). Thinking about spatial thinking: New typology, new assessments. Studying visual and spatial reasoning for design creativity.

[CR46] Oldakowski RK (2001). Activities to develop a spatial perspective among students in introductory geography courses. Journal of Geography.

[CR47] Orion N, Ben-Chaim D, Kali Y (1997). Relationship between earth-science education and spatial visualization. Journal of Geoscience Education.

[CR48] Peters M, Laeng B, Latham K, Jackson M, Zaiyouna R, Richardson C (1995). A redrawn Vandenberg and Kuse mental rotations test-different versions and factors that affect performance. Brain and Cognition.

[CR49] Philbeck JW, Behrmann M, Black SE, Ebert P (2000). Intact spatial updating during locomotion after right posterior parietal lesions. Neuropsychologia.

[CR50] Piburn MD, Reynolds SJ, McAuliffe C, Leedy DE, Birk JP, Johnson JK (2005). The role of visualization in learning from computer-based images. International Journal of Science Education.

[CR51] Rezvan PH, Lee KJ, Simpson JA (2015). The rise of multiple imputation: a review of the reporting and implementation of the method in medical research. BMC Medical Research Methodology.

[CR52] Richardson Anthony E., Montello Daniel R., Hegarty Mary (1999). Spatial knowledge acquisition from maps and from navigation in real and virtual environments. Memory & Cognition.

[CR53] Schinazi VR, Nardi D, Newcombe NS, Shipley TF, Epstein RA (2013). Hippocampal size predicts rapid learning of a cognitive map in humans. Hippocampus.

[CR54] Self CM, Gopal S, Golledge RG, Fenstermaker S (1992). Gender-related differences in spatial abilities. Progress in Human Geography.

[CR55] Shea DL, Lubinski D, Benbow CP (2001). Importance of assessing spatial ability in intellectually talented young adolescents: a 20-year longitudinal study. Journal of Educational Psychology.

[CR56] Shipley Thomas F., Tikoff Basil (2019). Collaboration, cyberinfrastructure, and cognitive science: The role of databases and dataguides in 21st century structural geology. Journal of Structural Geology.

[CR57] Strauss E, Sherman EMS, Spreen O (2006). A compendium of neuropsychological tests: Administration, norms, and commentary.

[CR58] Tobler WR (1994). Bidimensional regression. Geographical Analysis.

[CR59] Unsworth N, Heitz RP, Schrock JC, Engle RW (2005). An automated version of the operation span task. Behavior Research Methods.

[CR60] Uttal David H., Meadow Nathaniel G., Tipton Elizabeth, Hand Linda L., Alden Alison R., Warren Christopher, Newcombe Nora S. (2013). The malleability of spatial skills: A meta-analysis of training studies. Psychological Bulletin.

[CR61] Vandenberg SG, Kuse AR (1978). Mental rotations, a group test of three-dimensional spatial visualization. Perceptual and Motor Skills.

[CR62] Wai J, Lubinski D, Benbow CP (2009). Spatial ability for STEM domains: aligning over 50 years of cumulative psychological knowledge solidifies its importance. Journal of Educational Psychology.

[CR63] Weisberg SM, Newcombe NS (2016). How do (some) people make a cognitive map? Routes, places, and working memory. Journal of Experimental Psychology: Learning, Memory, and Cognition.

[CR64] Weisberg SM, Schinazi VR, Newcombe NS, Shipley TF, Epstein RA (2014). Variations in cognitive maps: understanding individual differences in navigation. Journal of Experimental Psychology: Learning, Memory, and Cognition.

[CR65] White IR, Royston P, Wood AM (2011). Multiple imputation using chained equations: issues and guidance for practice. Statistics in Medicine.

[CR66] Wilkinson GS, Robertson GJ (2006). WRAT 4: Wide Range Achievement Test.

